# Adjunctive use of physostigmine salicylate (Anticholium®) in perioperative sepsis and septic shock: study protocol for a randomized, double-blind, placebo-controlled, monocentric trial (Anticholium® per Se)

**DOI:** 10.1186/s13063-017-2231-x

**Published:** 2017-11-10

**Authors:** Johannes B. Zimmermann, Nadine Pinder, Thomas Bruckner, Monika Lehmann, Johann Motsch, Thorsten Brenner, Torsten Hoppe-Tichy, Stefanie Swoboda, Markus A. Weigand, Stefan Hofer

**Affiliations:** 1 Department of Anaesthesiology, University Hospital Heidelberg, Im Neuenheimer Feld 110, 69120 Heidelberg, Germany; 2 Pharmacy Department, University Hospital Heidelberg, Im Neuenheimer Feld 670, 69120 Heidelberg, Germany; 3 Institute of Medical Biometry and Informatics, University Hospital Heidelberg, Marsilius-Arkaden, Tower West, Im Neuenheimer Feld 130.3, 69120 Heidelberg, Germany; 4 Coordination Centre for Clinical Trials, University Hospital Heidelberg, Marsilius-Arkaden, Tower West, Im Neuenheimer Feld 130.3, 69120 Heidelberg, Germany

**Keywords:** Physostigma venenosum, Antilirium, Cholinesterase inhibitor, Continuous administration, Eserine, Eseroline, Intra-abdominal infection, Critically ill, Cholinergic anti-inflammatory pathway, Sequential Organ Failure Assessment (SOFA) score

## Abstract

**Background:**

Severe sepsis and septic shock remain a major challenge, even in modern intensive care. In Germany, about 68,000 patients die annually because of septic diseases, characterized by a complex systemic inflammatory response. Causal treatment of the underlying infection is essential for successful management of sepsis, but the course can be positively influenced by supportive and adjuvant measures. The cholinergic anti-inflammatory pathway (CAP) represents a new approach to adjunctive therapy of septic diseases and can be pharmacologically activated by the acetylcholinesterase inhibitor physostigmine (Anticholium®). Promising effects can be found in several in vitro and in vivo models of sepsis, such as a reduction in pro-inflammatory cytokines and improved survival.

**Methods:**

Anticholium® per Se is a randomized, double-blind, placebo-controlled, monocentric trial to assess whether the CAP can be transferred from bench to bedside. In this pilot study, 20 patients with perioperative sepsis and septic shock as a result of intra-abdominal infection are enrolled. According to randomization, participants are treated with physostigmine salicylate (verum group) or 0.9% sodium chloride (placebo group) for up to 5 days. The mean Sequential Organ Failure Assessment (SOFA) score during treatment and subsequent intensive care of up to 14 days is used as surrogate outcome (primary endpoint). Secondary outcome measures include 30- and 90-day mortality. An embedded pharmacokinetics and pharmacodynamics study investigates plasma concentrations of physostigmine and its metabolite eseroline. Further analyses will contribute to our understanding of the role of various cytokines in the pathophysiology of human sepsis. A computer-generated list is used for block randomization.

**Discussion:**

This randomized, controlled, monocentric trial investigates for the first time the adjunctive use of physostigmine (Anticholium®) in patients with perioperative sepsis and septic shock and may be a pivotal step toward the clinical use in this indication.

**Trial registration:**

EudraCT Number: 2012-001650-26 (entered 14 August 2012), ClinicalTrials.gov identifier: NCT03013322 (registered on 1 Jan 2017).

**Electronic supplementary material:**

The online version of this article (doi:10.1186/s13063-017-2231-x) contains supplementary material, which is available to authorized users.

## Background

### Background and rationale

In Germany, septic diseases kill approximately 68,000 patients each year [1]. About 280,000 (335 of 100,000) contract sepsis, approximately 115,000 of whom (138 of 100,000) experience severe sepsis or septic shock [1]. For those with severe sepsis, the probability of dying in hospital is about 44%, and increases to 59% for septic shock [1].

In view of these alarming figures, new approaches in treatment are required. One new treatment concept is based on the results of the group of Tracey [2].

In an in vitro test series with lipopolysaccharide (LPS)-treated human macrophage cultures, acetylcholine, the neurotransmitter of the parasympathetic nervous system, significantly inhibited the release of various pro-inflammatory cytokines, such as tumor necrosis factor (TNF)-α, interleukin (IL)-1β, IL-6, and IL-18, but not the anti-inflammatory cytokine IL-10 [2]. Suppression of TNF-α was reversed with α-bungarotoxin, but not with atropine, a specific muscarinic inhibitor [2]. Additionally, the peripheral vagus nerve was stimulated electrically, i.e., directly, in rats that had received the endotoxin intravenously. Stimulation of the parasympathetic nerve inhibited TNF-α synthesis in the liver and decreased peak serum TNF-α [2]. The decrease in blood pressure following endotoxemia was reduced significantly, and consequently, the progression from sepsis to severe sepsis and septic shock [2]. Thus, the inflammatory response can be influenced via α-bungarotoxin-sensitive nicotinic acetylcholine receptors.

These results have been confirmed by other groups in various sepsis models and have deepened our understanding of the cholinergic anti-inflammatory pathway (CAP) [3].

Taking into account the toxicity of the substances, concentrations used in test series, and ethical reservations about the direct electrical stimulation of the vagus nerve, the CAP must be accessible for patients in a different way.

Physostigmine salicylate is a peripherally and centrally acting inhibitor of acetylcholinesterase, and has been licensed and in clinical use for many years [4]. Acetylcholinesterase catalyzes the cleavage of acetylcholine to acetate and choline. Via decreased cleavage of the endogenous neurotransmitter, it thus alters acetylcholine levels. In an in vivo test series with mice that had undergone cecal ligation and puncture (a recognized sepsis model), we showed that physostigmine salicylate significantly inhibited the release of various cytokines (TNF-α, IL-1β, and IL-6); similar to the results of the group headed by Tracey [3]. Binding activity of nuclear factor (NF)-κB, a specific transcription factor, and activity of myeloperoxidase, a measure of pulmonary neutrophil invasion, was also attenuated [3]. The treatment diminished the decrease in blood pressure following infection, and lethality was reduced to the same extent as with nicotine [3]. Thus, the inflammatory response can be influenced by the use of physostigmine salicylate and this well-tolerated drug reduces the mortality in what is sometimes called the standard model for polymicrobial sepsis [5].

Pharmacological properties of physostigmine have been studied in animals including guinea pigs and of course rats, and humans [6]. The pyrroloindole alkaloid is predominantly metabolized in the liver and for a lesser part hydrolyzed by plasma esterases, with a respective half-life of 18–30 min. Metabolites are excreted with urine and feces [7].

Usually, physostigmine is administered by intravenous injection or short infusion. A few studies describe continuous infusion [4, 8, 9]. However, reliable pharmacokinetics and pharmacodynamics (PK/PD) data are still sparse, especially for patients with sepsis and septic shock. Note that the human subjects mentioned earlier were healthy volunteers [9] and some patients with Alzheimer’s disease [8]. In patients with sepsis and septic shock, however, the distribution, metabolism, and excretion of drugs are altered by complex and sometimes opposing changes and mechanisms including fluid extravasation, hemodynamic instability, and organ failure [10], possibly influencing pharmacodynamic effects [11].

This randomized, double-blind, placebo-controlled, monocentric pilot study on the use of physostigmine salicylate (Anticholium®) as an adjunctive measure in perioperative sepsis and septic shock is conducted to provide evidence of the effectiveness of physostigmine in humans - a clinical trial on an approved drug in a new indication.

Plasma concentrations of physostigmine and its metabolite eseroline are determined and a population pharmacokinetic model will be developed. Furthermore, cholinesterase activity is studied as a direct pharmacodynamic effect of physostigmine.

Further analyses will illuminate the role of various cytokines in the pathophysiology of human sepsis.

### Objectives

Anticholium® per Se evaluates the effect of treatment with physostigmine salicylate or drug-free placebo on the mean Sequential Organ Failure Assessment (SOFA) score (at least two individual values) during treatment and subsequent intensive care of up to 14 days in critically ill patients with perioperative sepsis and septic shock due to intra-abdominal infection, to assess whether the CAP can be transferred from bench to bedside.

## Methods: participants, interventions, and outcomes

### Trial design

Anticholium® per Se is a prospective, randomized (1:1), double-blind, placebo-controlled, monocentric pilot study. Only patients with perioperative sepsis and septic shock as a result of intra-abdominal infection are eligible. Participants are randomly assigned to one group that receives physostigmine salicylate (verum group), or to the other group that receives 0.9% sodium chloride (placebo group). The treatment period is at most 5 days, and follow-up is limited to 90 days. The mean SOFA score (at least two individual values) during treatment and subsequent intensive care of up to 14 days is used as surrogate outcome (primary endpoint). Secondary outcome measures include 30- and 90-day mortality. An embedded PK/PD study investigates plasma concentrations of physostigmine and its metabolite eseroline.

### Study setting

Anticholium® per Se is conducted in the Department of Anesthesiology, Heidelberg University Hospital. The Interdisciplinary Operative Intensive Care Unit (IOPIS) is a 16-bed ICU that admits a wide variety of surgical patients. This ICU handled 1556 admissions in the past 2 years, with an artificial ventilation ratio of approximately 60%. The executing clinic is monitored by the local Coordination Centre for Clinical Trials (KKS), which is responsible for study management and medical device safety. Data management and statistical analysis are provided by the Institute for Medical Biometry and Informatics (IMBI), Heidelberg.

### Eligibility criteria

Inclusion and exclusion criteria for participants are provided in Table 1.

**Table 1:** Eligibility criteria for participants

Inclusion criteria	Exclusion criteria
• Age 18–85 years • APACHE II score < 34 • Intra-abdominal infection ◦ findings of diffuse peritonitis or ◦ a circumscribed abscess • Perioperative sepsis and ◦ secure evidence of infection, ◦ clinically backed up or ◦ secured microbiologically ◦ ≥ 2 of the following four criteria: ▪ fever ≥ 38.0 °C or ▪ hypothermia ≤ 36.0 °C secured by rectal intravesical or intravascular measurement ▪ tachycardia ≥ 90/min ▪ tachypnea ≥ 20/min or ▪ hyperventilation secured by arterial blood gas analysis with PaCO_2_ ≤ 4.3 kPa or 33 mmHg or ▪ mechanical artificial respiration ▪ leukocytosis ≥ 12,000/mm^3^ or ▪ leukopenia ≤ 4000/mm^3^ or ▪ ≥ 10% immature neutrophils in the differential count • Shock (<24 h duration): necessary use of vasopressors despite adequate fluid resuscitation to keep systolic blood pressure ≥ 90 mmHg or mean blood pressure ≥ 70 mmHg • No more than one planned and/or one emergency procedure performed since admission (no repeated revisions) • No infaust prognosis of a primary or concomitant illness, expecting the death within the follow-up phase • No do-not-resuscitate order • Written informed consent of full-age patients/their legal guardian to participate (written consent, according to AMG)	• Known hypersensitivity to physostigmine salicylate, sodium metabisulfite, sodium EDTA, or any of the other ingredients of Anticholium® • Known contraindications [7] against Anticholium®: gangrene, coronary artery disease • Known absolute contraindications [7] against Anticholium®: myotonic dystrophy; depolarization block by depolarizing muscle relaxants; intoxication by “irreversibly acting” cholinesterase inhibitors; closed craniocerebral trauma; obstruction in the gastrointestinal tract (mechanical constipation); obstruction in the urinary tract (mechanical urinary retention) • Known relative contraindications [7] against Anticholium®: bronchial asthma; bradycardia; AV-conduction disturbances • Having undergone splenectomy • Having undergone solid organ transplantation • Positive pregnancy test, pregnancy, and lactation • Participation in another clinical trial, according to AMG or the follow-up phase of another study, according to AMG

*AMG* Medicinal Products Act, *APACHE* Acute Physiology And Chronic Health Evaluation, *AV* atrioventricular, *PaCO*_*2*_ partial pressure of carbon dioxide

### Interventions

Participants are enrolled within 24 h of onset of shock as defined in Table 1. Study medication is commenced within the following 2 h. The treatment group receives an infusion of 0.04 mg/kg physostigmine salicylate with a maximum dose of 4 mg. The infusion is administered at 0.4 mg/min (=1 mL/min = 60 mL/h). The initial dose is followed by a continuous infusion of 0.017 mg/min, i.e., 1 mg/h (=0.042 mL/min = 2.5 mL/h) for 2–5 days, i.e., 48–120 hours (treatment phase). The placebo group is treated with 0.9% sodium chloride.

The continuous infusion should not be interrupted; not even for surgery. If it is, the interruption is recorded in the case report form (CRF). Any interruption for > 90 min (five elimination half-lives > 18 min in humans) is considered a protocol violation, and the protocol violation is recorded in the CRF. Any interruption for > 180 min (ten elimination half-lives > 18 min in humans) is considered a discontinuation of trial medication. If the continuous infusion is interrupted for > 90 but < 180 min, the initial dose is repeated before the continuous infusion is resumed for the remaining time.

Criteria for discontinuing trial medication for an individual participant on the part of the investigator include occurrence of adverse events or delayed observation of violation of eligibility criteria that let it appear medically advisable.

The protocol does not make any provisions regarding interventions that are permitted or prohibited during the trial.

### Outcomes

The mean SOFA score (at least two individual values) during treatment and subsequent intensive care of up to 14 days is used as surrogate outcome in critically ill patients with perioperative sepsis and septic shock due to intra-abdominal infection.

Secondary measures include duration of artificial ventilation and intensive care, length of stay, 30- and 90-day mortality, arterial and central venous blood gas analyses, partial pressure of arterial oxygen/fraction of inspired oxygen (PaO_2_/FiO_2_), platelet count, leukocyte count, creatinine, urea, total bilirubin, C-reactive protein, prothrombin time, D-dimer, procalcitonin, IL-6, thrombin-antithrombin complex, mean blood pressure and use of vasopressors (frequency and duration), renal replacement therapy (frequency and duration), Glasgow Coma Scale (GCS) score, Acute Physiology And Chronic Health Evaluation (APACHE) II score, Simplified Acute Physiology Score (SAPS) II score, and the occurrence of side effects such as nausea or vomiting, clinically relevant changes in heart rate or blood pressure (mainly hypotension), and clinically relevant changes in airway resistance (mainly bronchiospasms as a result of hypersensitivity reactions to the sodium metabisulfite contained in the investigational medicinal product - spontaneous breathing: acute dyspnea or artificial ventilation: clinically relevant decline in respiratory volume at constant pressure settings, or clinically relevant incline in peak or inspiratory pressures at constant respiratory volumes).

Laboratory values, except IL-6 and thrombin-antithrombin complex, are determined during routine clinical examination in the inter-laboratory proficiency tested Central Laboratory (CL), or with a respective point-of-care device provided by the CL (for blood gas analyses). IL-6 and thrombin-antithrombin complex (from citrated plasma) are determined in the investigator’s laboratory using commercially available enzyme-linked immunosorbent assay (ELISA) kits. Moreover, additional serum samples are retained for further analyses at a later date.

Microbiological analyses of potential pathogens including susceptibility tests are performed at Heidelberg University Hospital, Department of Medical Microbiology.

For the PK/PD study, heparinized plasma samples are collected during treatment phase and on the following day (visits 1–8, see Table 2). Plasma concentrations of physostigmine and its metabolite eseroline are determined with a validated high-performance liquid chromatography (HPLC) method [12]. Cholinesterase activity is determined with ChE check mobile (Securetec, Neubiberg, Germany) [13] from remaining material drawn for routine blood gas analyses (arterial samples). The point-of-care device is dedicated to photometric measurement of acetyl- and butyrylcholinesterase activity in whole blood.

**Table 2:**
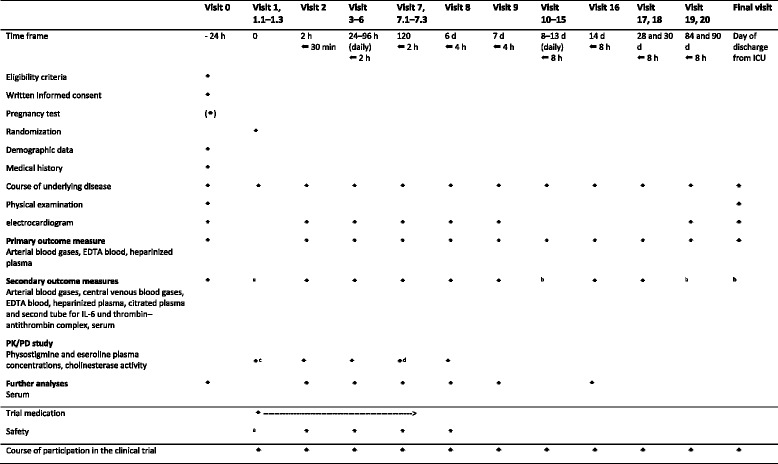
Flow chart of visits, interventions, and data collected for the outcomes

*AE* adverse events, *PD* pharmacodynamics, *PK* pharmacokinetics, *SAE* serious adverse events

^a^During administration of trial medication (initial dose), patients are continuously monitored by a physician attentive to the occurrence of side effects, AEs, and SAEs

^b^Marked visits do not include any additional collection of blood (no second tube for IL-6 and thrombin-antithrombin complex)

^c^Visit 1 including visit 1.1 with two blood samples [3 min after the start of the initial dose and at the end of the initial dose (±2 min)], visit 1.2 with three blood samples [10, 20, and 30 min after the start of continuous infusion (±2 min)], visit 1.3 with one blood sample [after 1 h (±10 min)]

^d^Visit 7 including visit 7.1 with one blood sample [at the end of the infusion period (±2 min)], visit 7.2 with three blood samples [10, 20, and 30 min after the end of the infusion period (±2 min)], visit 7.3 with two blood samples [after 1 and 2 h (±10 min)]

### Participant timeline

A flow chart of trial-specific procedures, assessments, and visits for participants is provided in Table 2.

The final visit is made on the day of discharge from ICU. In case of death, date of death is regarded as day of discharge from ICU and documented in the CRF accordingly. The final physical examination is omitted on the date of death. In all other cases, it is attempted to assess 30- and 90-day mortality, either from patient file or by telephone.

### Sample size

Anticholium® per Se is a pilot study precluding educated estimates. Primary results will be of great value in hypothesis generation. Thus, sample size was set for feasibility. With a number of 10 patients per treatment group and a sample size of 20 patients, a standardized effect (Cohen’s d) of 1.32 may be shown at a significance level of α = 5% with a power of 1 − β = 80%.

### Recruitment

All scheduled and all emergency admissions to the surgical clinic of the site are screened for inclusion and exclusion criteria. Patients operated on at the Surgical Clinic of the respective site who have to undergo subsequent surgery are repeatedly screened for inclusion and exclusion criteria. Between August 2003 and July 2008, the IOPIS handled 284 cases with intra-abdominal infection and sepsis, 206 of which had severe sepsis/septic shock [14]. In terms of figures, a little less than five patients with sepsis were thus cared for at IOPIS every month, a little more than three of which had severe sepsis/septic shock. Other clinical trials with similar eligibility criteria conducted by the Interdisciplinary Study Centre of the Departments of Anaesthesiology and Surgery, Surgical Clinic, Heidelberg University Hospital, during that period of time, were able to enrol approximately one subject every month.

## Methods: assignment of interventions

### Allocation

#### Sequence generation

For allocation of the participants, a randomization list was generated with SAS Proc Plan (SAS Institute Inc., Cary, NC, USA). The randomization sequence consists of unknown block sizes to balance the allocation of participants to groups if the trial is terminated before 20 participants have been reached. To further reduce biases due to other factors (apart from treatment) that may have changed over time [15], patients are treated according to the S-2 k guideline [16] of the German Sepsis Society (DSG) and German Interdisciplinary Association for Intensive Care and Emergency Medicine (DIVI). Block sizes are kept secret until unblinding.

#### Allocation concealment mechanism

The trial medication (verum and placebo) is prepacked in sequentially numbered containers according to the randomization list by Dr. Franz Köhler Chemie GmbH, the manufacturer. Participants are assigned order numbers and receive the medication in the corresponding containers.

#### Implementation

The randomization list was generated by the responsible biostatistician of the IMBI, who has no clinical involvement in the trial. Once the members of the study group have obtained informed consent, the investigator assigns the order numbers (patient number) and supplies the trial medication for application. However, participants are considered randomized with infusion of the initial dose.

### Blinding

The placebo has an identical pharmaceutical form and is externally indistinguishable when delivered, handled, e.g., drawn into the syringes, administered, or even spilled. The members of the study group are provided with ready-to-use, opaque 50-mL perfusor syringes with appropriate infusion lines containing the trial medication. The syringes handed to the members of the study group are labeled with the acronym Anticholium® per Se, EudraCT number, patient number, date of preparation, initials of the investigator or his representative, and the note Only for use within the clinical trial, and no other information, especially not for treatment group. The investigator and members of the study group, healthcare providers, data collectors, and participants are kept blinded during the clinical phase of the trial. To ensure blinding throughout the PK/PD study, plasma samples for the determination of physostigmine and eseroline plasma concentrations are immediately centrifuged, deep frozen, and stored at −70 °C until visit 16, to be analyzed later. The analyst is not involved in data acquisition with the respective patient any more. The assessment of cholinesterase activity is performed by a person not involved in further data acquisition from the respective patient.

Together with trial medication, the investigator is provided with a set of sealed envelopes, one per randomization number. These emergency envelopes contain information about the allocated intervention and may only be opened if it is medically indispensable to know what the participant has received. Once an envelope has been opened, date and reason must be documented and signed by the respective person. Whenever possible, the investigator is to consult with the deputy before unblinding.

## Methods: data collection, management, and analysis

### Data collection methods

All data collected in this trial will be recorded on standardized case report forms (CRF) which must ensure full documentation of all patient data required by the study protocol. Source data are available on worksheets or printed and diagnosed (e.g., electrocardiogram or laboratory results). The investigator is responsible for ensuring that all parts of the CRFs are filled in correctly. The CRFs are completed with pens, pencils are not allowed. Any change or correction to a CRF should be dated and initialed. Each CRF must be signed by the investigator at least once, the original is sent to the data management and a copy is retained at the study site.

### Data management

All protocol-required information collected during the trial must be entered by the investigator, or designated representative, in the case report form (CRF). The investigator, or designated representative, should complete the CRF pages as soon as possible after information is collected, preferably on the same day that a trial subject is seen for an examination, treatment, or any other trial procedure. Any outstanding entries must be completed immediately after the final examination. An explanation should be given for all missing data.

The completed CRF must be reviewed and signed by the investigator or by a designated sub-investigator. After keeping a copy at the trial center, the original CRF is sent to IMBI who is in charge of the data management within the trial.

In order to ensure that the database reproduces the CRFs correctly, the IMBI accomplishes a double entry of data to the statistical program SAS (SAS Institute, Inc., Cary, NC, USA). IMBI representatives will check completeness, validity, and plausibility of data using validating programs, which will generate queries. All validation rules will be predefined in a data validation plan. The investigator or the designated representatives are obliged to clarify or explain the queries. If no further corrections are to be made in the database it will be closed and used for statistical analysis. The data will be managed and analyzed according to the appropriate standard operation procedures (SOPs) valid in the IMBI.

According to section 13 of the GCP Ordinance [17], all important trial documents (e.g., CRFs) are archived for at least 10 years after completion of the clinical trial.

### Statistical methods

The primary endpoint is the mean SOFA score during treatment and subsequent intensive care of up to 14 days in the intention-to-treat (ITT) population. Anticholium® per Se being a pilot study missing values will not be imputed. The ITT population will include all randomized participants (full analysis population) from whom the primary outcome measure was obtained. The two-sided analysis of covariance (ANCOVA) model will include age as continuous covariate.

The per protocol population will consist of all participants from the ITT population without serious protocol violation. These include violations against eligibility criteria or departures from the treatment scheme, especially falling short of the minimum duration of administration. Exceeding the maximum duration of administration is not considered a serious protocol violation. Any interruption for > 90 min but ≤ 180 min and interruptions for source control of infection are not considered serious protocol violations, unless the continuous infusion is paused earlier than 90 min before the beginning or continued later than 90 min after the end of the intervention.

The χ2 test (categorical data) and further ANCOVA (continuous data) will be used for further descriptive analysis and secondary outcome measures, which will also be tabulated (measures of location and dispersion as appropriate for underlying empirical distribution). The Kaplan-Meier method and log-rank test will be used for survival analysis. Estimates of effect and 95% confidence intervals will be reported where possible and descriptive *P* values given. In addition, graphs are presented where possible.

Secondary analyses will include all participants from whom the primary outcome measure was obtained.

In the PK/PD study, plasma concentrations of physostigmine and eseroline are used to develop a population pharmacokinetic model for continuous intravenous administration. Possible structural models are examined and the impact of potential covariates (e.g., body weight, height, and gender) is investigated. Furthermore, PK/PD correlation of physostigmine concentrations and plasma cholinesterase activity is investigated.

## Methods: monitoring

### Data monitoring

Monitoring will be done by personal visits from a clinical monitor according to SOPs of the KKS. The monitor will review the entries into the CRFs on the basis of source documents. Frequency and details of monitoring will be defined in the monitoring manual. The investigator must allow the monitor to verify all essential documents and must provide support at all times to the monitor.

By frequent communications (letters, telephone, fax), the site monitor will ensure that the trial is conducted according to the protocol and regulatory requirements.

### Harms

Adverse events are defined as per International Conference on Harmonisation of Technical Requirements for Registration of Pharmaceuticals for Human Use (ICH): untoward medical occurrences in participants administered the treatment and which do not necessarily have causal relationships with this treatment [18]. AEs [including serious AEs (SAEs)] are recorded in the CRF. SAEs result in death or are life-threatening, require prolongation of existing hospitalization, result in persistent or significant disability or incapacity, or are otherwise medically relevant. SAEs are recorded in dedicated forms and reported to the KKS Safety Officer by fax, who forwards the forms to the investigator or his representative for re-assessment within 48 h. The re-assessment also addresses the question whether AEs or adverse reactions were expected. AEs or adverse reactions, the nature or severity of which are not consistent with the summary of product characteristics are regarded suspected unexpected SAEs or serious adverse reactions (SUSAR). They are subject to expedited reporting [18, 19] to the ethics committee, the Federal Institute for Drugs and Medical Devices (BfArM), and investigators within 15 days (8 days if resulting in death or being life-threatening). Furthermore, any overdose of the investigational medicinal product is reported to the sponsor representative within 24 h.

### Auditing

Regular audits by the sponsor are not intended. For the purpose of onsite inspection or audit, the competent authorities or KKS may require access to all source documents, CRF, and other trial-related records. The investigator must ensure availability of these documents and support the work at any time.

## Ethics

Described procedures for conducting, analysis, and documentation of Anticholium® per Se are meant to ensure that all parties involved abide by the principles of Good Clinical Practice (GCP) [17, 19] and those stipulated in the Declaration of Helsinki [20]. The conducting takes place in accordance with local statutory and implementing provisions. The provisions of the Medicinal Products Act (AMG) and the GCP Ordinance (GCP-V) are complied with [17, 21].

### Research ethics approval

Prior to the beginning of the clinical trial, the study protocol, the patient information and informed consent, and all other required documents (see GCP-V section 7 [17]) were submitted to the competent ethical review committee and the corresponding higher federal authority (BfArM).

### Protocol amendments

Changes to the protocol are made in writing and require the approval of all signatories of the protocol. Subsequent amendments also require a positive assessment from the competent ethics committee and must be authorized by the BfArM [17].

### Confidentiality

Data collected are handled in accordance with the provisions of the Federal Data Protection Act (BDSG) [22].

During the clinical trial, participants are solely identified by a distinct reference number (i.e. randomization number). For storage on a computer, the provisions of the BDSG [22] are abided by. Data are handled with strict confidentiality. For protection of these data, organizational measures are taken to prevent disclosure to unauthorized third parties. The relevant rules of the country-specific data legislation are complied with.

### Ancillary and post-trial care

Upon completion, termination or exclusion patients are treated according to the S-2 k guideline [16] of German Sepsis Society (DSG) and German Interdisciplinary Association for Intensive Care and Emergency Medicine (DIVI).

## Discussion

### Risks, side effects, exposures, advantages and disadvantages for participants

Physostigmine salicylate has been licensed and in clinical use for many years [4]. The drug is approved for the treatment of postoperative disorders, such as central anticholinergic syndrome and delayed postoperative awakening and shivering. In 2006, the Interdisciplinary Operative Intensive Care Unit (IOPIS), Heidelberg University Hospital, the center where the present pilot study is being conducted, approximately 180 patients were treated with physostigmine without relevant side effects. A low risk of side effects in clinical use was reported in previous studies [4, 23–26, 27]. In a clinical trial with 50 patients undergoing lower abdominal surgery, half of whom were treated late intraoperatively with physostigmine 1.5 mg intravenously and immediately postoperatively with physostigmine 1 mg/h over 24 h, there was a higher incidence of postoperative nausea and vomiting, but no other side effects such as bradycardia, hypotension, or bronchospasm [4]. In Anticholium® per Se, according to random allocation, participants receive an infusion of 0.04 mg/kg physostigmine salicylate with a maximum dose of 4 mg or a drug-free placebo. The infusion rate should be 0.4 mg/min (=1 mL/min = 60 mL/h). The infusion dose expressed in mg/kg body weight corresponds to the recommended dose for treatment of postoperative awakening disorders (0.04 mg/kg). The maximum dose exceeds the maximum single dose (2 mg) for the treatment of postoperative awakening disorders. According to the summary of product characteristics (SmPC), further injections should be administered no earlier than 5–20 min after the effect of the first injection can be assessed adequately [7]. Patients with postoperative awakening disorders can receive a maximum single dose of 2 mg according to the SmPC. Repeated injections may be performed after at least 5 min and patients can receive a maximum dose of 4 mg in 7 min (physostigmine is injected slowly intravenously at about 1 mg/min).

Although the maximum dose exceeds the maximum single dose for the treatment of postoperative awakening disorders, the infusion rate is 0.4 mg/min (=1 mL/min = 60 mL/h). This can be considered as a single dose of 2 mg physostigmine salicylate over 5 min and re-injection after 5 min of not more than 2 mg physostigmine salicylate for 5 min. For this infusion regimen, we can assume a similar risk of side effects as for the use of physostigmine salicylate for treatment of postoperative awakening disorders.

In Anticholium® per Se enrolled participants receive a continuous infusion of 0.017 mg/min physostigmine salicylate for 2–5 days (verum group) or a 0.9% sodium chloride (placebo group). This regimen corresponds to the rate of continuous infusion in a previous clinical trial [4]. For the duration of infusion, there may be a similar risk of side effects as in the previous clinical trial (higher incidence of postoperative nausea and vomiting, but no other side effects). Because of the seriousness of sepsis, and in particular, severe sepsis and septic shock, as well as the possible underlying disorder and comorbidity, the majority of participants in Anticholium® per Se are sedated and ventilated. In these patients, the stomach is relieved by a gastric tube. The higher incidence of postoperative nausea and vomiting found in the previous clinical trial does not impose an additional risk.

Only a few trial-related procedures include the withdrawal of blood samples. These measures represent a minor additional burden but no additional risk.

### Justification for enrolment of participants not capable of giving consent

Besides causal treatment (focal restoration, and use of antibiotics), there is the symptomatic, i.e., diagnosis-related therapy of sepsis, severe sepsis, and septic shock. Although using all supportive (hemodynamic stabilization, and artificial ventilation) and adjunctive (low-dose use of hydrocortisone) measures, causal treatment is not always successful and the lethality remains high. Early treatment is crucial to disease progression. The treatment is probably only successful if the corresponding medication is administered within a few hours after the first symptoms. However, early treatment does not guarantee success. For patients with severe sepsis, the probability of dying in hospital is approximately 44%, and for patients with septic shock, it is approximately 59% [1]. With regard to these alarming figures, the current clinical trial is designed to investigate a new therapeutic approach, namely, physostigmine salicylate. The majority of the patients affected are sedated and given artificial ventilation. Even before being sedated, affected patients must be regarded incapable of giving consent due to intra-abdominal infection, inflammatory response, and severe pain. Furthermore, after waking from an artificial coma or general anesthesia required for the surgery to focus remediation, we consider that patients should be regarded as incapable of giving consent for at least another 24 h. For this period of time, informed consent to participate in Anticholium® per Se is given by a legal guardian until the affected patients are capable of consent (Additional file 1).

### Trial status

Anticholium® per Se is an ongoing trial that has not completed patient recruitment.

## Additional file

Additional file 1: SPIRIT 2013 Checklist: recommended items to address in a clinical trial protocol and related documents*. (DOC 122 kb)

## Abbreviations

AE: Adverse event
AMG: Medicinal Products Act
ANCOVA: Analysis of covariance
APACHE: Acute Physiology And Chronic Health Evaluation
BDSG: Federal Data Protection Act
BfArM: Federal Institute for Drugs and Medical Devices
CAP: Cholinergic anti-inflammatory pathway
CL: Central Laboratory
CRF: Case report form
DIVI: German Interdisciplinary Association for Intensive Care and Emergency Medicine
DSG: German Sepsis Society
ELISA: Enzyme-linked immunosorbent assay
FiO_2_: Fraction of inspired oxygen
GCP-V: GCP Ordinance
GCS: Glasgow Coma Scale
HPLC: High-performance liquid chromatography
ICH: International Conference on Harmonisation of Technical Requirements for Registration of Pharmaceuticals for Human Use
IL: Interleukin
IMBI: Institute for Medical Biometry and Informatics
IOPIS: Interdisciplinary Operative Intensive Care Unit
ITT: Intention-to-treat
KKS: Coordination Centre for Clinical Trials Heidelberg
LPS: Lipopolysaccharide
NF: Nuclear factor
PaO_2_: Partial pressure of arterial oxygen
PD: Pharmacodynamics
PK: Pharmacokinetics
SAE: Serious AE
SAPS: Simplified Acute Physiology Score
SmPC: Summary of product characteristics
SOFA: Sequential Organ Failure Assessment
SOP: Standard operation procedure
SUSAR: Suspected unexpected SAE or serious adverse reaction
TNF: Tumor necrosis factor

## References

[1] Fleischmann C, Thomas-Rueddel DO, Hartmann M, Hartog CS, Welte T, Heublein S, Dennler U, Reinhart K (2016). Hospital incidence and mortality rates of sepsis. Dtsch Arztebl Int.

[2] Borovikova LV, Ivanova S, Zhang M, Yang H, Botchkina GI, Watkins LR, Wang H, Abumrad N, Eaton JW, Tracey KJ (2000). Vagus nerve stimulation attenuates the systemic inflammatory response to endotoxin. Nature.

[3] Hofer S, Eisenbach C, Lukic IK, Schneider L, Bode K, Brueckmann M, Mautner S, Wente MN, Encke J, Werner J (2008). Pharmacologic cholinesterase inhibition improves survival in experimental sepsis. Crit Care Med.

[4] Beilin B, Bessler H, Papismedov L, Weinstock M, Shavit Y (2005). Continuous physostigmine combined with morphine-based patient-controlled analgesia in the postoperative period. Acta Anaesthesiol Scand.

[5] Dejager L, Pinheiro I, Dejonckheere E, Libert C (2011). Cecal ligation and puncture: the gold standard model for polymicrobial sepsis?. Trends Microbiol.

[6] Triggle DJ, Mitchell JM, Filler R (1998). The pharmacology of physostigmine. CNS Drug Rev.

[7] Dr. Franz Köhler Chemie GmbH. SmPC (Fachinformation) Anticholium® Injektionslösung. 2011.

[8] Asthana S, Raffaele KC, Berardi A, Greig NH, Haxby JV, Schapiro MB, Soncrant TT (1995). Treatment of Alzheimer disease by continuous intravenous infusion of physostigmine. Alzheimer Dis Assoc Disord.

[9] Furey ML, Pietrini P, Alexander GE, Mentis MJ, Szczepanik J, Shetty U, Greig NH, Holloway HW, Schapiro MB, Freo U (2000). Time course of pharmacodynamic and pharmacokinetic effects of physostigmine assessed by functional brain imaging in humans. Pharmacol Biochem Behav.

[10] Roberts JA, Lipman J (2009). Pharmacokinetic issues for antibiotics in the critically ill patient. Crit Care Med.

[11] De Paepe P, Belpaire FM, Buylaert WA (2002). Pharmacokinetic and pharmacodynamic considerations when treating patients with sepsis and septic shock. Clin Pharmacokinet.

[12] Pinder N, Zimmermann JB, Hofer S, Brenner T, Weigand MA, Gubbe U, Hoppe-Tichy T, Swoboda S (2015). Revival of physostigmine - a novel HPLC assay for simultaneous determination of physostigmine and its metabolite eseroline designed for a pharmacokinetic study of septic patients. Clin Chem Lab Med.

[13] Worek F, Baumann M, Pfeiffer B, Aurbek N, Thiermann H. Mobiler cholinesterase-schnelltest zur felddiagnostik einer organophosphat-exposition im vollblut. Wehrmedizinische Monatsschrift. 2011;4.

[14] Lichtenstern C, Zimmermann JB, Rahbari NN, Uhle F, Kerber S, Weismuller K, Hofer S, Walter V, Bruckner T, Weitz J, Weigand MA (2011). Patients suffering due to complicated peritonitis may not benefit from splenectomy: clinical data from a retrospective study. J Surg Res.

[15] Altman DG, Bland JM (1999). Statistics notes. Treatment allocation in controlled trials: why randomise?. BMJ.

[16] Reinhart K, Brunkhorst FM, Bone HG, Bardutzky J, Dempfle CE, Forst H, Gastmeier P, Gerlach H, Grundling M, John S (2010). Prevention, diagnosis, therapy and follow-up care of sepsis: 1st revision of S-2k guidelines of the German Sepsis Society (Deutsche Sepsis-Gesellschaft e.V. (DSG)) and the German Interdisciplinary Association of Intensive Care and Emergency Medicine (Deutsche Interdisziplinare Vereinigung fur Intensiv- und Notfallmedizin (DIVI)). Ger Med Sci.

[17] GCP Ordinance (GCP-Verordnung, GCP-V). 2004. http://www.gesetze-im-internet.de/gcp-v/index.html. Accessed 14 Oct 2016.

[18] ICH E2A: International Conference on Harmonisation Tripartite Guideline: clinical safety data management: definitions and standards for expedited reporting. 1994. https://www.ich.org/fileadmin/Public_Web_Site/ICH_Products/Guidelines/Efficacy/E2A/Step4/E2A_Guideline.pdf. Accessed 14 Oct 2016.

[19] ICH E6(R1): International Conference on Harmonisation Tripartite Guideline: Good Clinical Practice. 1996. https://www.ich.org/fileadmin/Public_Web_Site/ICH_Products/Guidelines/Efficacy/E6/E6_R1_Guideline.pdf. Accessed 14 Oct 2016.

[20] World Medical Association (2013). World Medical Association Declaration of Helsinki: ethical principles for medical research involving human subjects. JAMA.

[21] Medicinal Products Act (Arzneimittelgesetz, Gesetz über den Verkehr mit Arzneimitteln, AMG). 1976. http://www.gesetze-im-internet.de/amg_1976/index.html. Accessed 14 Oct 2016.

[22] Federal Data Protection Act (Bundesdatenschutzgesetz, BDSG). 1990. https://www.gesetze-im-internet.de/bdsg_1990/index.html. Accessed 14 Oct 2016.

[23] Paraskeva A, Papilas K, Fassoulaki A, Melemeni A, Papadopoulos G (2002). Physostigmine does not antagonize sevoflurane anesthesia assessed by bispectral index or enhances recovery. Anesth Analg.

[24] Petersson J, Gordh TE, Hartvig P, Wiklund L (1986). A double-blind trial of the analgesic properties of physostigmine in postoperative patients. Acta Anaesthesiol Scand.

[25] Kesecioglu J, Rupreht J, Telci L, Dzoljic M, Erdmann W (1991). Effect of aminophylline or physostigmine on recovery from nitrous oxide-enflurane anaesthesia. Acta Anaesthesiol Scand.

[26] Paraskeva A, Staikou C, Diamadis M, Siafaka I, Fassoulaki A (2005). Anesthesia with 1.5 minimum alveolar concentration sevoflurane is not altered by physostigmine as measured by bispectral and clinical indices. J Clin Anesth.

[27] Rohm KD, Riechmann J, Boldt J, Schollhorn T, Piper SN (2007). Do patients profit from physostigmine in recovery from desflurane anaesthesia?. Acta Anaesthesiol Scand.

[28] International Committee of Medical Journal Editors (ICMJE) Recommendations: Defining the Role of Authors and Contributors. http://www.icmje.org/recommendations/browse/roles-and-responsibilities/defining-the-role-of-authors-and-contributors.html. Accessed 14 Oct 2016.

[29] Steiner T, Walter-Sack I, Taupitz J, Hacke W, Strowitzki T (2008). Ethical and legal aspects of including patients unable to consent in acute therapy studies. Example of a medication study for the treatment of intracerebral hemorrhage--the Heidelberg procedure. Dtsch Med Wochenschr.

